# The effect of mesenchymal stem cells-derived exosomes on the prostate, bladder, and renal cancer cell lines

**DOI:** 10.1038/s41598-022-23204-x

**Published:** 2022-12-03

**Authors:** AhmadReza Rezaeian, Fatemeh Khatami, Saeed Heidari Keshel, Mohammad Reza Akbari, Akram Mirzaei, Keykavos Gholami, Reza Mohammadi Farsani, Seyed Mohammad Kazem Aghamir

**Affiliations:** 1 Shahid Beheshti University of Medical Sciences, Tehran, Iran; 2 Urology Research Center, Tehran University of Medical Sciences, Tehran, Iran; 3 Department of Tissue Engineering and Applied Cell Sciences, School of Advanced Technologies in Medicine, Shahid Beheshti University of Medical Sciences, Tehran, Iran; 4 Women’s College Research Institute, Women’s College Hospital, University of Toronto, Toronto, Canada; 5 Institute of Medical Sciences, Faculty of Medicine, University of Toronto, Toronto, Canada

**Keywords:** Cancer, Stem cells

## Abstract

We aimed to explain the role of mesenchymal stem cells (MSC-exosomes) on gene expressions of epithelial to mesenchymal transition (EMT), angiogenesis, and apoptosis. Four different cell lines were employed, including ACHN, 5637, LNCaP, and PC3, as well-known representatives for renal, bladder, hormone-sensitive, and hormone-refractory prostate cancers, respectively. Cell lines were exposed to diverse concentrations of mesenchymal stem cells-derived exosomes to find IC50 values. Percentages of apoptotic cells were evaluated by Annexin/P.I. staining. Micro Culture Tetrazolium Test assessed proliferative inhibitory effect; and prostate biomarker (KLK2), EMT (E-cadherin and Snail), angiogenesis genes (VEGF-A/VEGF-C), apoptosis genes (BAX/BCL2, P53) and Osteopontin variants (OPNa/b, and c) mRNA levels were studied by realtime PCR method. All 5637, LNCaP, and PC3 following treatment with exosomes illustrated specific responses with changes in expression of different genes. The increased TP53 and decreased BCL2 expressions were seen in 5637, LNCaP, and PC3. In PC3, OPNb and OPNc have raised more than P53; in LNCap, the increase was in VEGF-c. In 5637 cells, more than TP53 and BCL2 changes, two other genes, VEGFa and B.A.X., have decreased, suggesting exosomes’ anti-apoptotic and anti-angiogenic effects. The kidney tumor cell line saw no significant gene expression change in ten targeted genes. MSC-exosomes therapy has augmented some interesting antitumor effects on prostate, bladder, and kidney cancer cell lines. This effect which originates from exosomes’ potency to persuade apoptosis and prevent the proliferation of cancer cells simultaneously, was more substantial in bladder cancer, moderate in prostate cancer, and mild in renal cancer.

## Introduction

The National Cancer Institute’s report shows that three urological cancers are among the top ten frequent cancers. Prostate, bladder, kidney, and renal pelvis cancers ranked second, sixth, and eighth common cancer types^1^. Approximately 248,500 new prostate cancer cases are estimated in 2021, accounting for about 13% of all new cancer cases in the U.S.A.^1^. The other two urological cancers are less common. However, their incidence is still high, with 83,730 new bladder cancers and 76,080 new kidney and renal pelvis cancers^1^. Of every three male individuals, one is diagnosed with one of these three cancers in their lifetime.

Although some developments in cancer treatments have occurred in the past decade, cancer treatment is still challenging, and advanced urological cancers require more effective therapies^2,3^. Cell-based treatments have been promising, but the unfavorable immunological reaction could complicate their usage^4^. Recent growing evidence showed that stem cells could play a therapeutic role via secreting various paracrine factors, mediated by some tiny functional vesicles^5–7^. In 1981, it was shown that the small vesicles released by different cells are isolable^8^. Several biologic components and chemicals are anticancer reagents^9–11^. These tiny vesicles were later named exosomes by Johnstone et al.^12,13^. Exosomes showed advantages over other cellular therapies, which resulted in graft versus host disease (GVHD) and emboli formation^14^. Exosomes are a subgroup of extracellular products with diameters ranging from 50 to 120 nm and cup-shaped morphology^14^. It has been shown that cancer cells secrete more exosomes than healthy cells^15^. These small vesicles generally contribute to tumor formation, growth, progression, invasiveness, and the ability to escape from the host immune system^3,16^. Exosomes can change epithelial-mesenchymal transition (E.M.T.), angiogenesis, and apoptosis^17^. E.M.T. process is a crucial step in cancer invasion and metastasis. E.M.T. phenotype is related to dysregulation of E-cadherin and Snail genes^18–20^. Angiogenesis provides more blood flow enriched in oxygen and nutritious substances required for progression^21^.

Blocking cancer crosstalk via depleting its exosomes from the circulatory system is outlined in many studies as an advanced method for tumor treatment^8^. Diluting these troublesome vesicles by adding healthy exosomes could be another possible creative technique. This manuscript evaluates the effect of MSC-exosomes on ACHN, 5637, LNCaP, and PC3 as representatives for renal, bladder, hormone-sensitive prostate, and hormone-refractory prostate cancers, respectively, as used in former studies^22–24^.

## Materials and methods

### Cell lines

5637 Cell line as primary bladder tumor, ACHN Cell line as metastatic renal adenocarcinoma, LNCaP Cell line as metastatic prostate cancer, and PC3 Cell line as prostate adenocarcinoma (grade IV) with ATCC number HTB-9, CRL-1611, CRL-10995, and CRL-1435 respectively prepared and cultured the (National Bank of Iran Cell Reserves under the name of Pasteur Institute-Tehran). All four cell lines cultured in Gibco DMEM medium (Carlsbad, CA) supplemented with 10% Gibco F.B.S. (Carlsbad, CA), Gibco penicillin (1000 U/ml), and Gibco streptomycin (100 µg/ml) (B.R.L.) and then incubated at 37 °C, 90% humidity with 5% CO_2_ incubator. During our testing, we confirmed that all methods followed the ABCAM’s protocol (https://www.abcam.com/) according to relevant guidelines and regulations.

### Preparation, isolation, and culture of adipose tissue-derived mesenchymal cells

In this in vitro study, 10 g of abdominal adipose tissue was used to isolate mesenchymal stem cells (M.S.C.) from a referred patient for liposuction after signing the written informed consent. The research was run under the Tehran University of Medical Sciences ethical committee (IR.TUMS.MEDICINE.REC.1400.159). The fat sample taken in saline buffer phosphate solution containing the antibiotic penicillin–streptomycin was transferred to a cell culture laboratory under sterile conditions. After several washing steps with saline buffer phosphate and the physiological serum, the tissue was cut into small pieces. By collagenase I, adipose tissue was digested, and mesenchymal stem cells were extracted from this tissue.

First, 1.5 mg of collagenase I enzyme was added for each gram of fat and incubated for 60 min at 37 °C. Then, it was centrifuged for 10 min at 2000 rpm. Finally, the cell sediment in DMEM (Dulbecco’s Modified Eagle’s Medium) culture medium with 1% penicillin and streptomycin, 10% F.B.S. (fetal bovine serum) was moved to a T75 cell culture flask and incubated at 37 °C with 5% carbon dioxide (CO_2_) and 90% humidity. The cell culture medium was changed every four days. The trypsin cells were then isolated from the flask and ready for use when they reached the third or fourth passage stage^25^.

### Cell characterization based on cell markers

Flow cytometry was used to determine M.S.C.s isolated from adipose tissue. In this method, 250,000 cells were used. The 75 T flask containing these cells in passage three was centrifuged after rinsing with PBS and trypsinized. After counting with 2–3 ml of PBS solution, centrifuged again, and the supernatant discarded. The cells were homogenized using 250 μl PBS. 50 μl of the suspension and 5 μl of each conjugated monoclonal antibodies to the fluorescent dyes were added to each tube. In parallel, the cells were exposed to the isotype control antibody. Then, the tubes were incubated for 40 min at 4 °C and finally analyzed by using flow cytometry^26^.

Antibodies used for fat stem cell examination checked the expression of CD90-FITC and CD105-FITC proteins and the lack of expression of CD45-FITC and HLA-DR proteins. Antibodies used to examine exosomes target CD9, CD63, and CD81 proteins. After evaluating cells, the DMEM culture medium was replaced with free serum exosome. After 48 h, the supernatant of the cells was collected, and the EXOCIB kit (Cib Biotech Co. Iran) was used to extract the exosomes.

### Exosome isolation and determination

We isolated the Exosomes from the M.S.C.s media by using the Exocib isolation kit. Exocib isolation kit had two reagents, A and B. According to the manufacturer’s protocols, first of all, we removed cellular debris, then collected the cell media and centrifuged the falcon (10 min, 3500 rpm by SIGMA 3-18KHS heated, refrigerated table top centrifuge, Germany). Then the collected supernatant was vortexed with reagent A (ratio 5:1), then vortexed for 10 min, and incubated for 24 h at refrigerator temperature. The combination was centrifuged (45 min, 2800 rpm), and the exosome plate was washed with 100 µl of reagent B. Ultimately, the exosome plate was saved at − 80 °C. Exosomes’ determination was carried on via employing flow cytometry and scanning electron microscopy.

**Figure 1:**
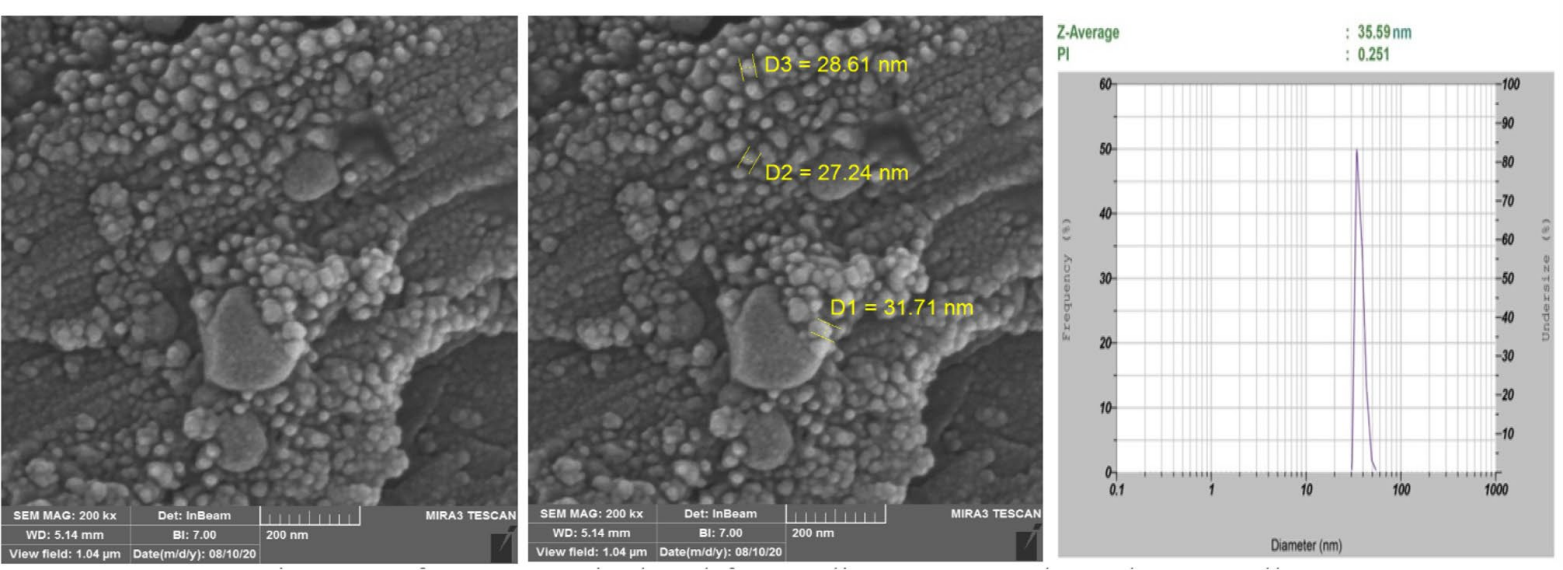
S.E.M. images of exosomes isolated from adipose mesenchymal stem cells. The DLS results also demonstrated that the Z-Average of vesicles is 35.59 nm with a PdI (polydispersity index) of 0.251.

To confirm the extracted Exosome, we used electron microscope imaging (MIRA3 TESCAN) with the following characteristics: S.E.M. magnification and resolution: 200kx, Det.in beam (detector is inside the column), B.I.: 7.00, working distance: 5.14 mm, view filed: 1.04 µm and Scale bar: 200 nm (Fig. 1).

### Micro culture tetrazolium test

LNCaP, ACHN, 5637, and PC3 cell lines were implanted in a plate (4 × 103 cells/well-96 well). All four cells were exposed to extracted exosomes and incubated for 3different times, 24/48 and 72 h. The rate of cell proliferation was assessed using the M.T.T. method. After these times, Hiperion NanoDrop (Medizintechnik GmbH & Co.KG, Germany) read the OD of cell viability at 570 nm following the manufacturer's protocol. The MTT test (three replications) was completed to evaluate IC50 (half-maximum inhibitory concentration).

### Apoptosis assay and D.N.A. cell cycle flow cytometry analysis

To evaluate the Apoptosis assay, PC3, LNCaP, 5637, and ACHN cancer cell lines were implanted in the six-well plates separately and incubated for 48 h in the presence and absence of exosomes. Induction of apoptosis by exosomes evaluated by Annexin V-FITC staining test. To assess the D.N.A. cell cycle assay, after 48 h, PC3, LNCaP, 5637, and ACHN cancer cell lines were exposed to exosomes, and the cells were washed with cold PBS and fixed with cold ethanol (70%). After one night, washed with PBS twice, all four cancer cell lines and incubated with RNase I, then stained with 500μL propidium iodide dye (PI) By BD Flow cytometer, separated PC3, LNCaP, 5637, ACHN cancer cell lines, and Flowjo software 7.6.1. (https://flowjo.software.informer.com) analyzed data according to the manufacturer’s procedure. The calculated values of sub-G1 are considered apoptotic values.

### R.N.A. isolation, cDNA synthesis, and real-time PCR

All four cancer cell lines, ACHN, LNCaP, 5637, and PC3, were exposed to exosomes and investigated for expression of some essential genes like *BCL2, B.A.X.*, and *P53* (apoptosis genes), *Osteopontin (O.P.N.)*, and *vascular endothelial growth factor or VEGF* (angiogenesis genes) isoforms, *KLK2* (prostate biomarker), and expression of *Snail, E-cadherin (E.M.T. marker)*. R.N.A. was extracted via the Roche TRIzol, and complementary D.N.A.s (cDNAs) were reverse transcribed using a Takara cDNA synthesis kit (According to the kit manufacturer’s instructions). To estimate the expression of mentioned genes, the 2^−ΔΔCT^ method is with the RT-PCR cycler QIAGEN’s machine. The nucleotide sequence of the primers in this study is consistent with the previous study^20,27,28^.

### Statistical analysis

All documents were available as means ± S.D. of the three duplicate measurements. T-test and ANOVA analyses were used to evaluate the outcomes.

### Ethical considerations

All patients signed the written informed consent, and the Tehran University of Medical Sciences ethical committee (IR.TUMS.MEDICINE.REC.1400.159) approved the study.

## Results

**Figure 2:**
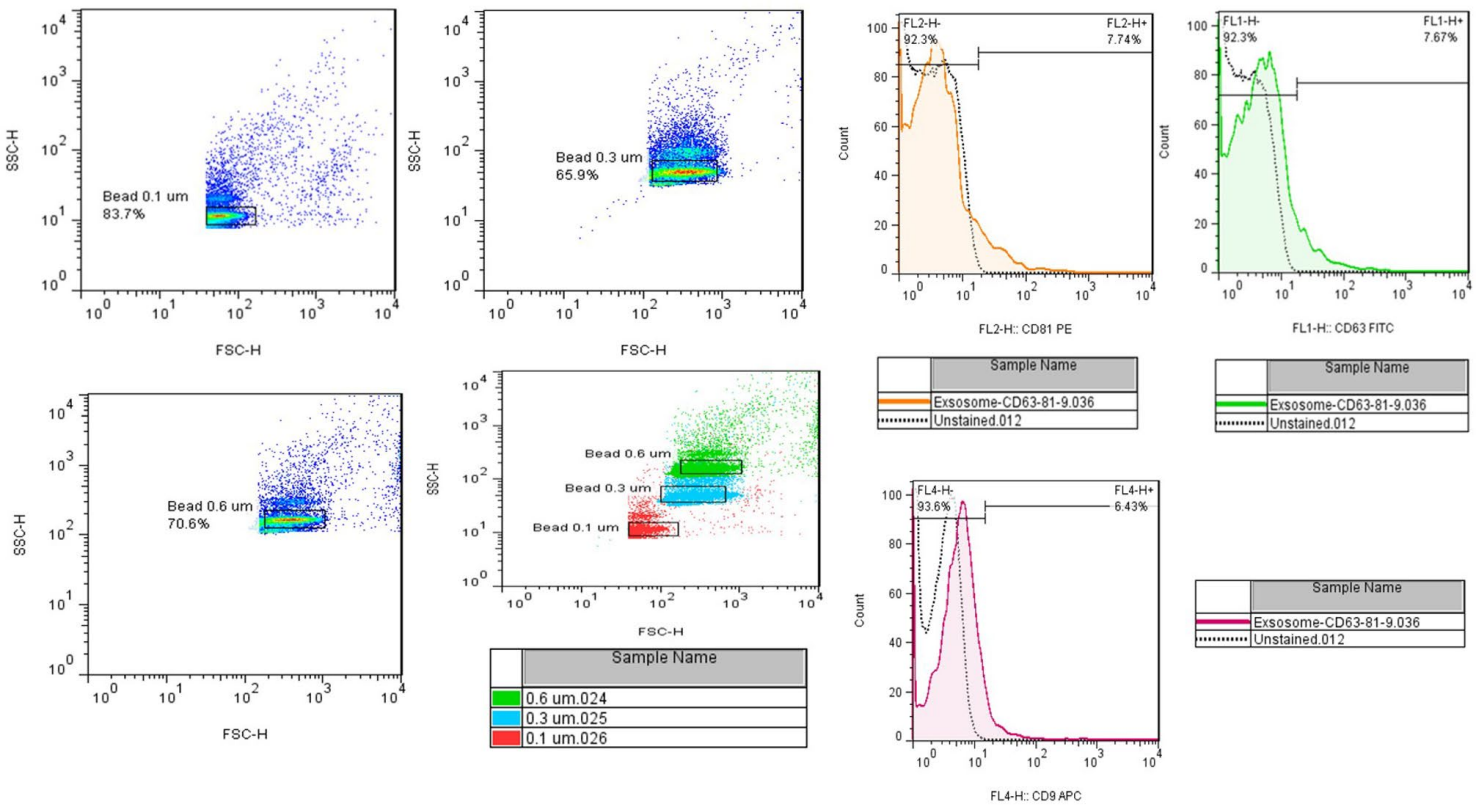
Antibodies used to examine exosomes target CD9, CD63, and CD81 proteins.

**Figure 3:**
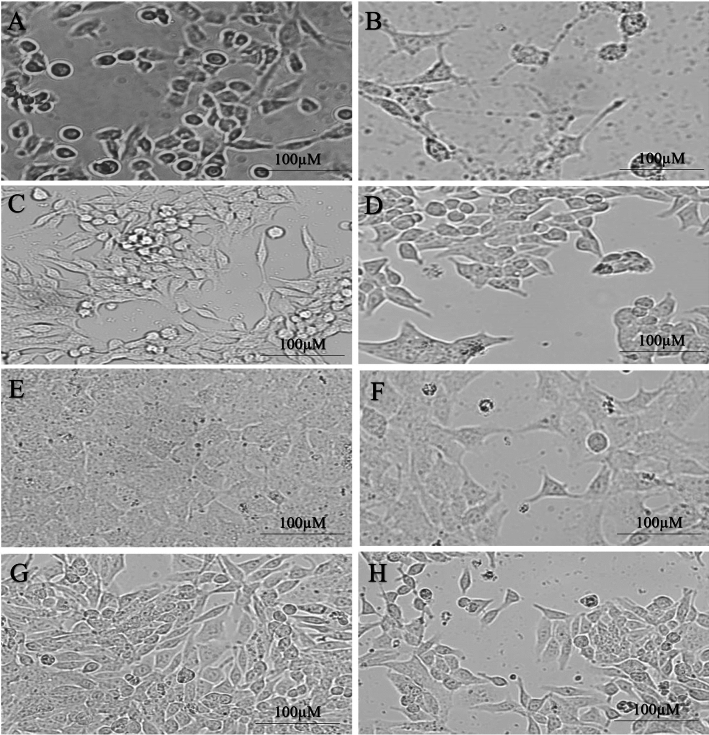
Light microscopic images of prostate, bladder, and kidney cancer cell lines. Untreated and treated PC3 cells with exosomes (**A**, **B**), untreated and treated LNCaP cells with exosomes (**C**, **D**), untreated and treated 5637 cells with exosomes (**E**, **F**), and finally, untreated and treated 5637 cells with exosomes (**G**, **H**) are shown.

The extracted exosomes were confirmed by the relevant antibodies (CD9, CD63, and CD81) through flow cytometry and apparently, through electron microscope images (Figs. 1, 2). The morphology of prostate, bladder, and kidney cancer cell lines treated with exosomes after 48 h have been shown using light microscopy (Fig. 3).

### Exosomes inhibit cell proliferation

**Figure 4:**
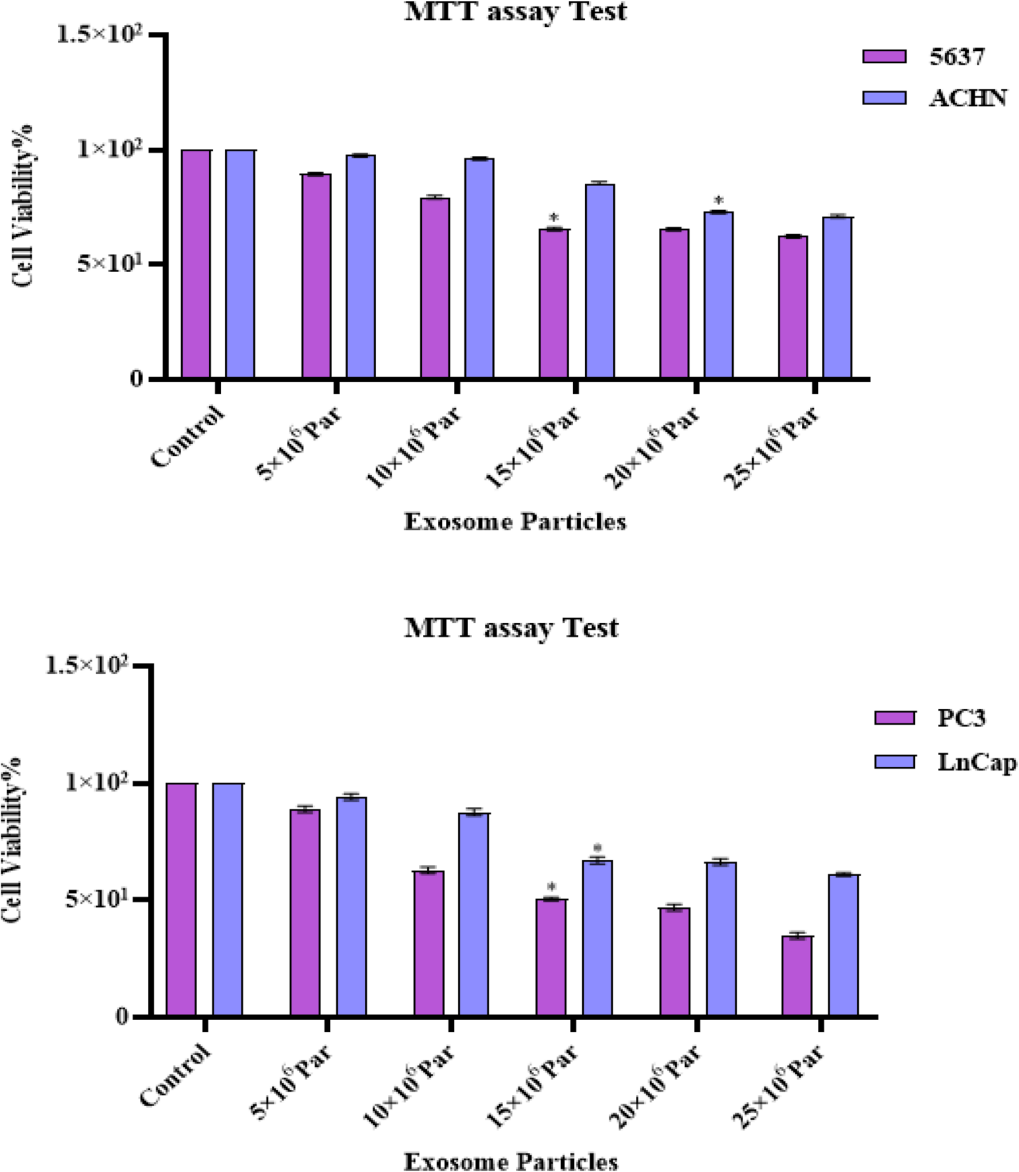
Effects of exosomes in different concentrations (0–25 × 10^6^ Par) on cell proliferation. The anti-growth effect of exosomes was measured by M.T.T. assay following 24, 48, and 72 h in 5637, ACHN, LNCaP, and PC3 cell lines. IC50 pharmaceutical doses of 15 × 10^6^ Particle in PC3, LNCap, and 5637 cell line and 15 × 10^6^ Particle in ACHN cell line were determined. Data showed that exosomes' anti-proliferative impact reduces cell viability with a dose- and time-dependent manner.

The cytotoxic effect of exosomes (0–25 × 10^6^ Par) was investigated in PC3, LNCaP, 5637, and ACHN cell lines for 24/48 and 72 h (Fig. 4). According to the results obtained in this study, IC50 values of exosomes were 15 × 106 Particles for PC3, LNCaP, and 5637 cell lines; and 20 × 106 Particles for ACHN cell line. The results presented that exosomes had a significant cytotoxic effect on all four target cell lines in a dose-dependent and time-dependent manner.

### Exosomes can stimulate apoptosis

**Figure 5:**
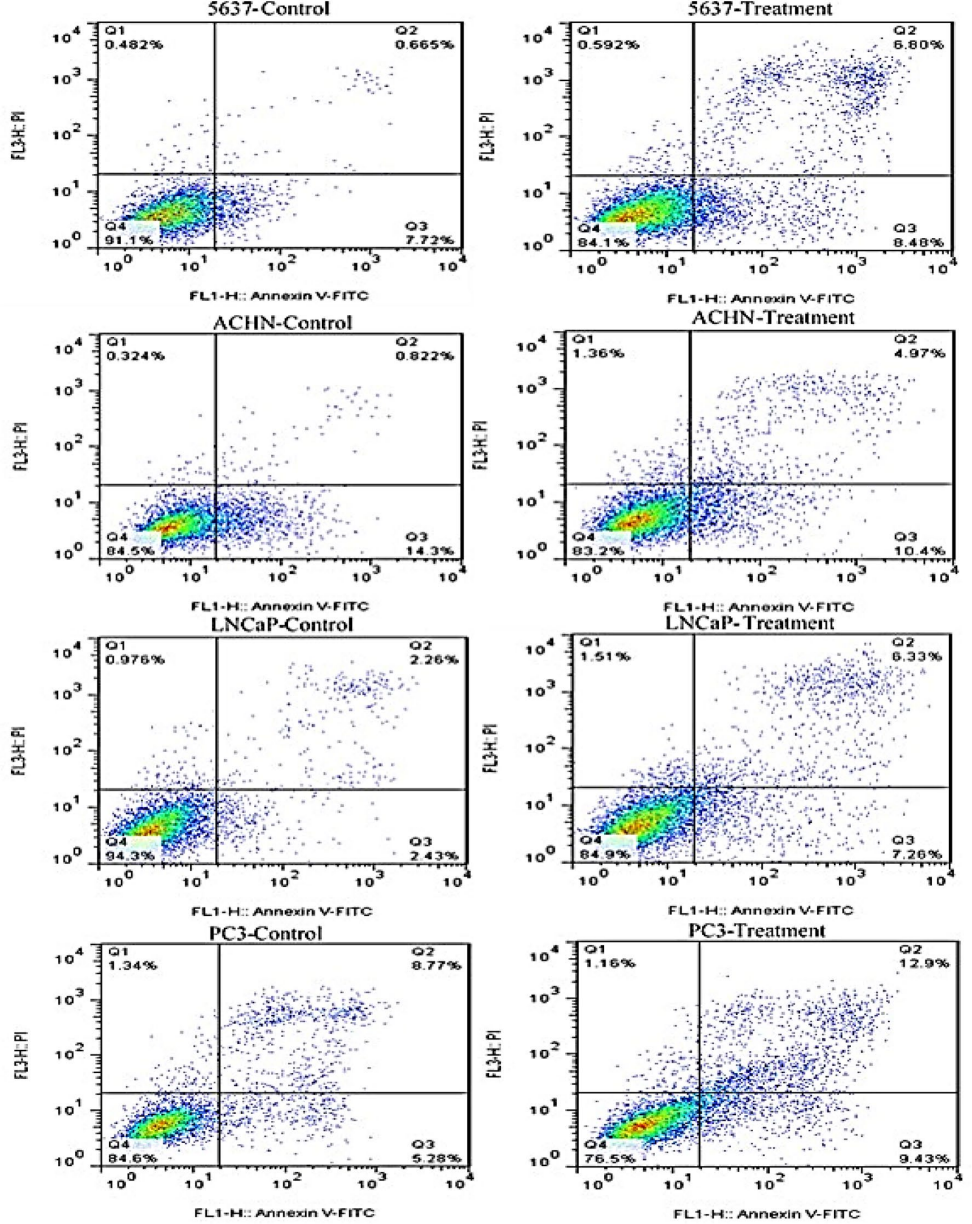

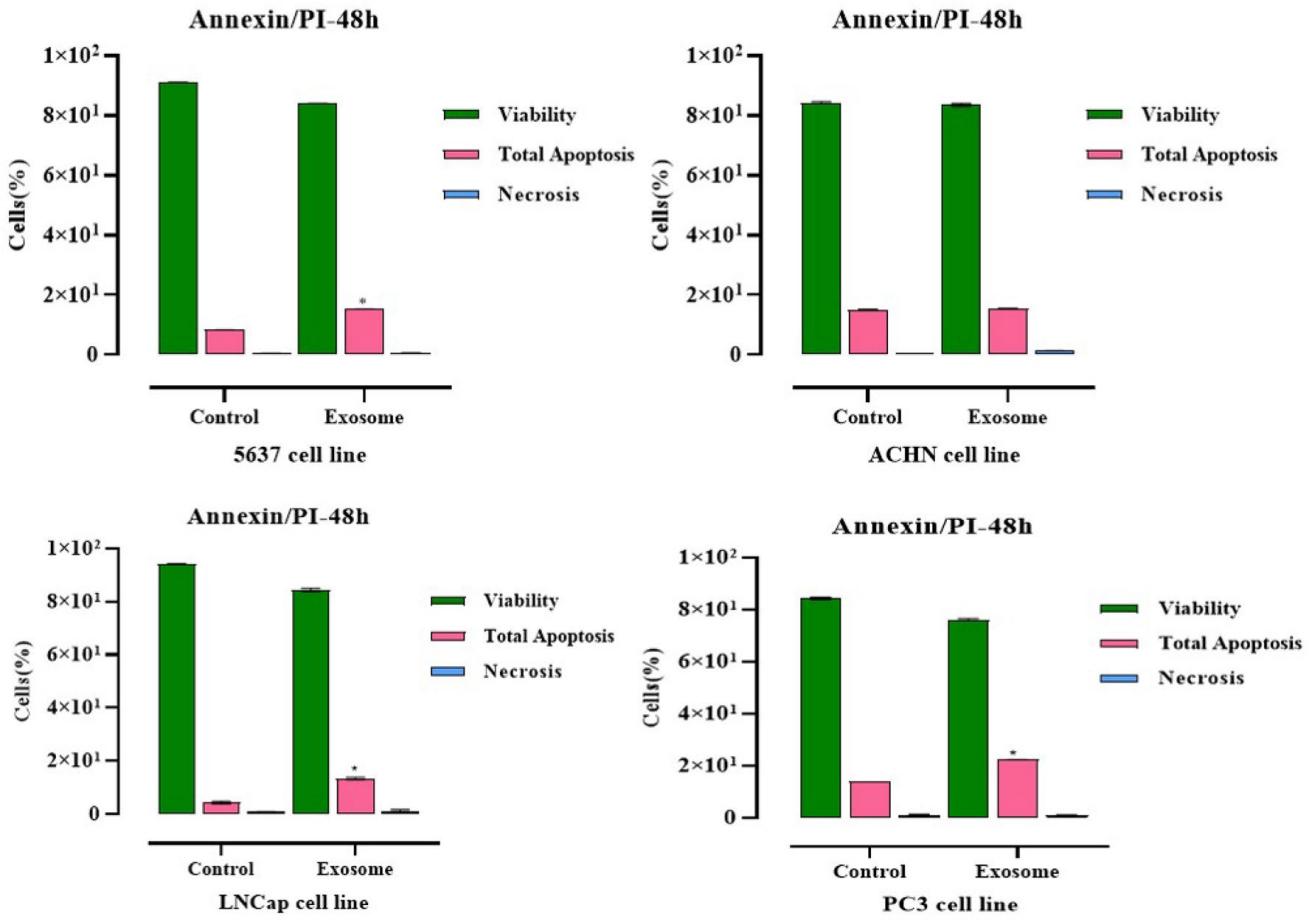
Flow cytometric analysis of LNCaP, PC3, ACHN, and 5637 cells apoptosis using Annexin-V-Flous. The lower left quadrant shows live cells, the lower right quadrant shows early apoptotic cells, the upper right quadrant shows late apoptotic cells, and the upper left quadrant shows necrotic cells. Results regarding induced apoptosis of PC3, LNCaP, 5637 and ACHN cancer cells untreated and treated with Exosome. Statistical significance was defined at *P < 0.05, **P < 0.01, and ***P < 0.001 compared to the corresponding control.

To investigate the effect of exosomes on apoptosis of PC3, ACHN, LNCaP, and 5637 cancer cell lines, we used Annexin/P. I method with flow cytometry. The outcomes were an apoptotic increase in four cancer cell lines treated with exosomes compared to the control group (Fig. 5). We recognized an induction in total apoptosis (early and late apoptotic cells), Annexin V-positive/PI-negative as Annexin+/P.I, and the lowest percentage of necrosis (Annexin-/PI+) in cured cells while at the end-stage of apoptotic cells are Annexin V/PI++ showed as Annexin V-FITC positive/PI-positive, compared with the control group in all four targeted cancer cell lines.

### Exosomes encourage sub-G1 and G1 arrest in prostate, bladder, and kidney cancer cell lines

**Figure 6:**
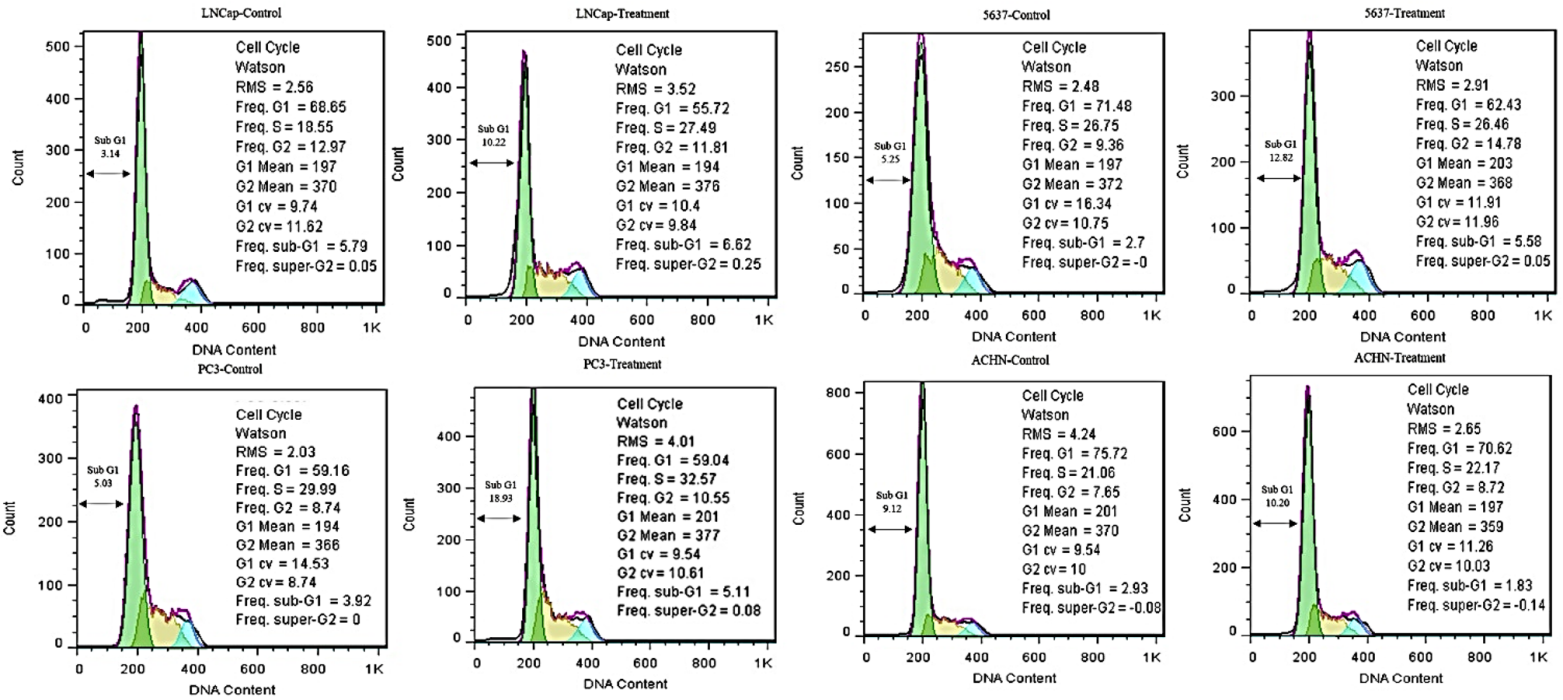
Cell cycle analysis of LNCaP, PC3, 5637, and ACHN cancer cell lines. DNA content of LNCaP, PC3, 5637, and ACHN cancer cell lines was assessed after exposed with exosomes by PI staining. The cell cycle analysis detected G1 area increased in LNCaP, PC3, 5637, and ACHN cell lines when treated with exosomes. The percentages of cells at the G2 phase were concurrently reduced in all treated cells. Therefore, exosomes apparently induce sub G1/G1 arrest in LNCaP, PC3, 5637, and ACHN cell lines.

Flow cytometry was used to study the induction of cell cycle arrest and the cell cycle of PC3, ACHN, LNCaP, and 5637 cancer cell lines treated with exosomes (Fig. 6).

The 5637, PC3, LNCap, and ACHN cells had a rise in sub-G1 phase cells (as apoptotic phase) following the exposure of cells with exosomes (5.25–12.82%), (5.03–18.93%), (3.14–10.22%) and (9.12–10.20%) besides the reduction in the following cell cycle phases, respectively. The outcomes of 5637, PC3, ACHN, and LNCap cancer cells showed that exosome-treated cells arrest the cell cycle in the G1 phase compared to the control group (71.48–62.43%), (59.16–59.04%), (68.65–55.72%) and (75.72–70.62%), respectively (Fig. 6).

### The impact of exosomes on gene expression

**Figure 7:**
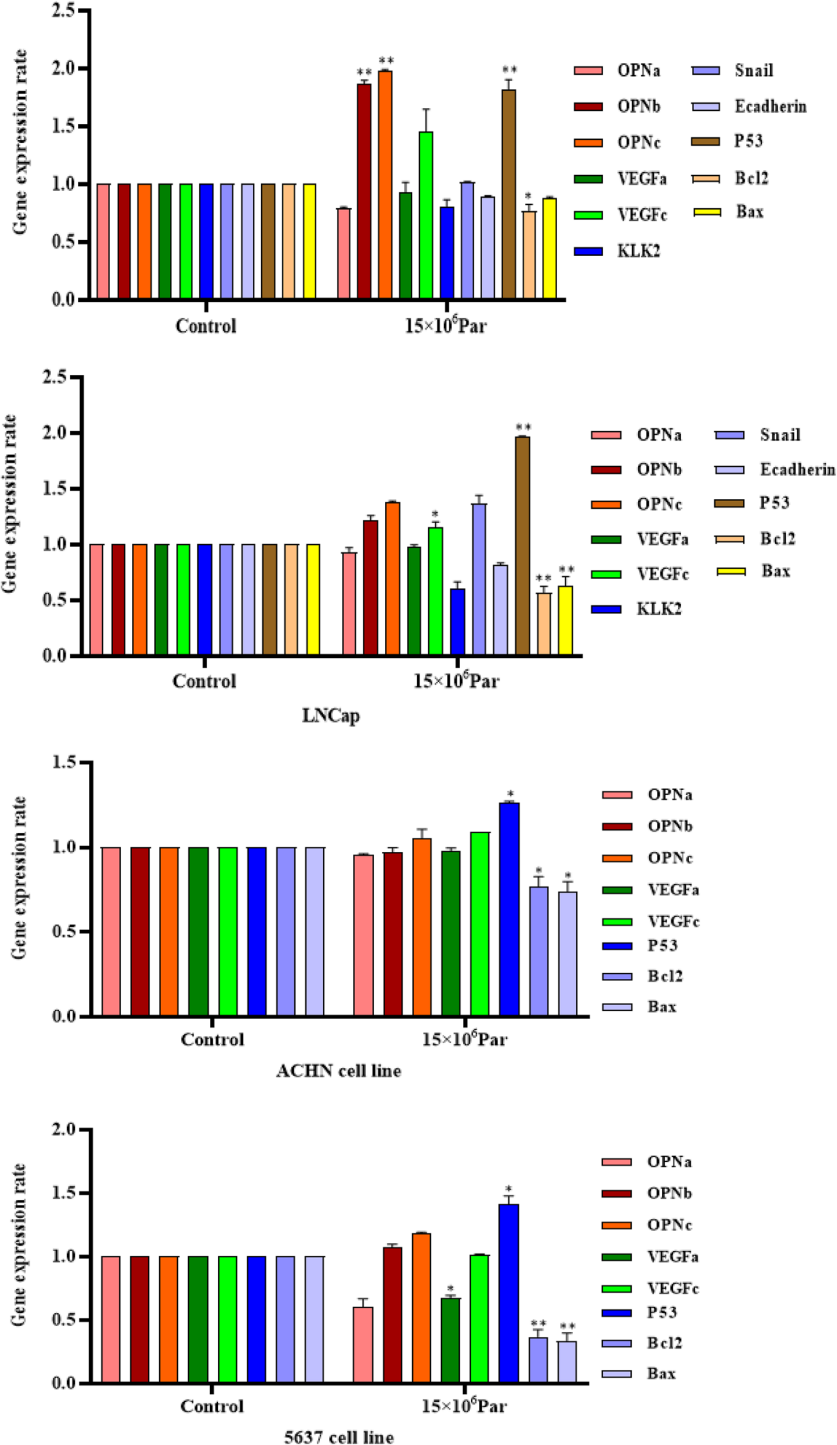
Results of PC3, LNCaP, 5637, and ACHN cancer cells exposed with exosomes on expression of selected genes after 48 h. Values are given as mean ± S.D. of three independent experiments. Statistical significance was defined at *P < 0.05, **P < 0.01, and ***P < 0.001 compared to the corresponding control.

It was observed that exosomes had the same synergistic apoptotic effect in LNCaP, PC3, and 5637 but not in ACHN cells by an increase of *P53* and a decrease of the *BCL2* gene expression. In PC3 cell lines, *OPNa, OPNc,* and *P53* gene expression increased after exosome treatment, contrary to *the BCL2* decrease. In another cell line of prostate cancer, LNCaP, the impact of exosomes was highlighted as the increase of *VEGF-c* and *P53* gene expression and reduction of *BCL2* and *BAX* gene expression. The most significant antitumor effect of exosomes was in bladder tumor cell line 5637 through TP53 gene expression increase simultaneously with a reduction in *VEGF-a/BAX*/*BCL2* genes. No significant gene expression changes of the ten investigated genes were seen for the kidney tumor cell line in ACHN cells (Fig. 7).

## Discussion

Our result indicated that exosomes could have an anticancer effect in PC3, LNCaP, ACHN, and 5637 by increasing cell apoptosis, necrosis and inhibiting cell proliferation. We showed that exosomes had the same synergistic apoptotic effect in LNCaP, PC3, and 5637 but not in ACHN cells by an increase of P53 and decrease of the BCL2 gene expression in exosomes-treated PC3, 5637, and LNCaP cancer cell lines.

The anticancer therapeutic prospects of MSC-exosomes in several tumors have been considered recently^29^. Recent studies have defined contradictory observations of MSC-derived exosomes' effect on cancer cells in which some suggested tumor growth and metastasis inducer, but others reported preventing tumor cell growth. Our data showed MSC-derived exosomes as an apoptotic factor in tumor cell lines of PC3, LNCaP, ACHN, and 5637. At the same time, it was demonstrated that secreted exosomes from cancer cell lines HeLa, PC3, MCF-7, Panc-1, and DLCL2 contain inhibitors of apoptosis. MSC-derived exosomes can fight with the tumor cells against modifications that frequently happen in the environment around a tumor^30^.

Furthermore, exosomes carry small noncoding RNAs called micro-RNAs playing role in posttranscriptional gene expression modulation^31^. It is shown that noncoding micro-RNA (mir-143) present in human bone marrow M.S.C. (BMSC)-derived exosomes can stop cell migration and metastasis in human prostate cancer. The mir-143 is a down regulator of the Trefoil factor 3 (TFF3) gene^32^. A study by Lázaro‐Ibáñez E. and colleagues showed that extracellular vesicles (E.V.s) originated from prostate cancer cell lines. Human plasma samples contain double-stranded genomic D.N.A. fragments with some mutations be considered as the biomarkers in both cancer detection and prognosis. Different fragmented genomic D.N.A. is found in subpopulations of prostate cancer E.V.s like apoptotic bodies, microvesicles, and exosomes^33–35^. Provided preclinical data indicates that Adipose-derived stromal cells (A.S.C.) prevent prostate cancer cell growth, causing prostate cancer cell apoptosis with reduced activity of BCL2L1 by miR-145, so it can be a new and favorable therapeutic strategy in patients with prostate cancer^36^. It has also been suggested that BMSC-derived exosomal miR-9-3p repressed bladder cancer progression through Endothelial cell-specific molecule 1 (ESM1) downregulation, presenting a novel therapeutic target for bladder cancer therapy^37^.

In our study, the Annexin V-FITC staining assay indicated the increasing apoptosis, necrosis, and inhibition of cell proliferation in all four target tumor cell lines when exposed to MSC-derived exosomes. Bot gene expression profile showed that 5637 was the most responsive tumor cell line to exosome therapy because both TP53 gene expression increased and VEGF-a gene expression decreased simultaneously. Zheng et al. showed that exosomal lncRNA brain cytoplasmic 200, brain cytoplasmic R.N.A. 1, was substantially upregulated in urinary exosomes from patients with a bladder tumor linked to lymph node metastasis^38^. Also, BCYRN1 increases VEGFC and its receptor (VEGF‐C/VEGFR3) signaling in bladder cancer patients with lymph node metastatic.

So, BCYRN1 can be a therapeutic target for patients with bladder cancer. Treatment of low (5637) and high (T24) grade bladder cancer cell lines with T24 tumor cell-derived exosomes can trigger cell proliferation and activation of Akt and ERK pathways^39^. A long noncoding R.N.A. is called Urothelial cancer associated 1 (RNA-UCA1) increases in bladder cancer and regulates the expression of several genes participating in tumorigenesis. It was shown by Xue M. et al. that RNA-UCA1 can increase bladder tumor growth and development^40^. MKP1 is a transcriptional target of p53. Cai X and colleagues showed that Exosome–transmitted microRNA‐133b reduced bladder cancer proliferation by an increase of DUSP1^41^.

Our data indicated both TP53 increase and BCL2 decrease in two prostate cancer cells, PC3 and LNCaP. The potential for creating new blood vessels from the pre-existing vessels of menstrual stem cells (MenSCs)-secreted exosomes on the PC3 cell line was assessed, and it was shown that MenSCs-derived exosomes could block tumor angiogenesis^42^. Yu et al. unexpectedly discovered that prostate cancer-derived exosomes are essential facilitators of bone homeostasis and osteoclastic lesions that stimulate tumor growth in bone^43^. Prostate-specific G-protein coupled receptors (PSGR) in prostate cancer are related to poor survival (O.S.). Exosomal PSGR triggers tumor cell migration, metastasis, stemness, and E.M.T. and reshapes LNCaP and RWPE-1 cells^44^. Elham et al. in vitro study showed that exosomes could enhance tumor size and serum P.S.A. level when xenograft-bearing mice have processed DU145 cell-derived exosomes intravenously^45^. They suggest that irrespective of androgen receptor phenotype, prostate tumor cells-derived exosomes considerably boost various mechanisms promoting prostate cancer progression^45^.

Yang Lin et al. reported that exosomes could stimulate tumor expansion and proliferation and prevent apoptosis in ACHN cell lines. Removal of the exosomes from the microenvironment of renal cancer or inhibition of its function can be new strategies for the treatment of renal cancer^46^. Several genetic biomarkers are presented in kidney tumors^47,48^. However, no significant change was seen in our study in all ten genes investigated. Based on Zhang L et al. study, renal cancer 786-0 cells-derived exosomes significantly promoted angiogenesis via upregulation of *VEGF* expression in human umbilical vein endothelial cells (HUVECs), which may be prompted by the decreasing of hepatocyte cell adhesion molecule^49^. It was shown that exosomes secreted by HEK293 (human embryonic kidney cells) and HT-1080 (fibrosarcoma) could suppress the growth and proliferation of *p53*-deficient cells^50^. So, Exosomes transfer p53 proteins between cells and can decrease cell proliferation and cell growth in p53-negative cells^50^. However, our data showed no significant gene expression change of *the P53* gene in ACHN cells as the kidney tumor cell line.

## Conclusion

We have employed mesenchymal stem cells derived exosomes for treating four urology human cancer cell lines. Our results demonstrated that this novel, safe method of cancer therapy could be effective in vitro, mainly on gene expression bases. In other words, exosome treatment ultimately can trigger various pathway modulation in apoptosis, angiogenesis, and E.M.T. processes, which could efficiently hinder tumor progression in bladder, prostate, and renal cancers, with bladder cancer as the best responder to this new cancer therapy.

## Data Availability

All information, data, and photos are all provided through the manuscript, and additional will be provided if requested.
