# Correction: The effect of mesenchymal stem cells-derived exosomes on the prostate, bladder, and renal cancer cell lines

**DOI:** 10.1038/s41598-025-33309-8

**Published:** 2026-02-18

**Authors:** AhmadReza Rezaeian, Fatemeh Khatami, Saeed Heidari Keshel, Mohammad Reza Akbari, Akram Mirzaei, Keykavos Gholami, Reza Mohammadi Farsani, Seyed Mohammad Kazem Aghamir

**Affiliations:** 1 Shahid Beheshti University of Medical Sciences, Tehran, Iran; 2 Urology Research Center, Tehran University of Medical Sciences, Tehran, Iran; 3 Department of Tissue Engineering and Applied Cell Sciences, School of Advanced Technologies in Medicine, Shahid Beheshti University of Medical Sciences, Tehran, Iran; 4 Women’s College Research Institute, Women’s College Hospital, University of Toronto, Toronto, Canada; 5 Institute of Medical Sciences, Faculty of Medicine, University of Toronto, Toronto, Canada

Correction to: *Scientific Reports* 10.1038/s41598-022-23204-x, published online 03 December 2022

The original version of this Article contained an error in Figure 3 where the incorrect image was included in panel A. The original Figure 3 and accompanying legend appear below.

**Fig. 3:**
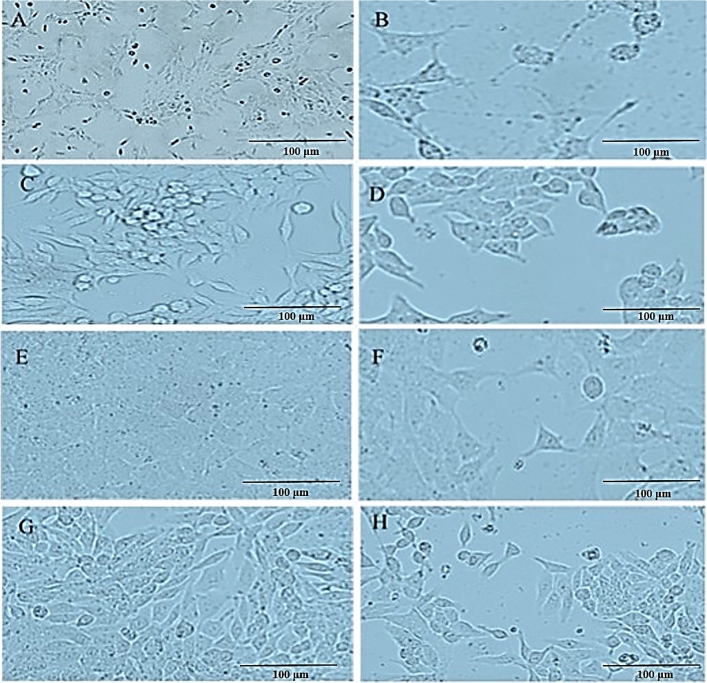
Light microscopic images of prostate, bladder, and kidney cancer cell lines. Untreated and treated PC3 cells with exosomes (**A**, **B**), untreated and treated LNCaP cells with exosomes (**C**, **D**), untreated and treated 5637 cells with exosomes (**E**, **F**), and finally, untreated and treated 5637 cells with exosomes (**G**, **H**) are shown.

The original Article has been corrected.

